# Behavioural and emotional issues among primary school pupils with congenital colour vision deficiency in the Federal Territory of Kuala Lumpur, Malaysia: A case-control study

**DOI:** 10.12688/f1000research.17006.1

**Published:** 2018-11-21

**Authors:** Belina Anne William M Thomas, Sharanjeet Kaur, Mohd Izzuddin Hairol, Mahadir Ahmad, Lei Hum Wee

**Affiliations:** 1 Optometry & Vision Science Programme, School of Healthcare Sciences, Faculty of Health Sciences, Universiti Kebangsaan Malaysia, Jalan Raja Muda Abdul Aziz, Kuala Lumpur, 50300, Malaysia; 2 Health Psychology Programme, School of Healthcare Sciences, Faculty of Health Sciences, Universiti Kebangsaan Malaysia, Jalan Raja Muda Abdul Aziz, Kuala Lumpur, 50300, Malaysia; 3 Health Education Programme, School of Healthcare Sciences, Faculty of Health Sciences, Universiti Kebangsaan Malaysia, Jalan Raja Muda Abdul Aziz, Kuala Lumpur, 50300, Malaysia

**Keywords:** behavioural problem, emotional problem, Child Behaviour Checklist/ ages 4-18, colour vision, congenital colour vision deficiency, colour blind, primary school pupil, quantitative method

## Abstract

**Background**: Congenital colour vision deficiency (CCVD) is an untreatable disorder which has lifelong consequences. Increasing use of colours in schools has raised concern for pupils with CCVD. This case-control study was conducted to compare behavioural and emotional issues among age, gender and class-matched pupils with CCVD and normal colour vision (NCV).

**Methods**: A total of 1732 pupils from 10 primary schools in the Federal Territory of Kuala Lumpur were screened, of which 46 pupils (45 males and 1 female) had CCVD. Mothers of male pupils with CCVD (n=44) and NCV (n=44) who gave consent were recruited to complete a self-administered parent report form, Child Behaviour Checklist for Ages 4-18 (CBCL/ 4-18) used to access behavioural and emotional problems. The CBCL/ 4-18 has three broad groupings: Internalising, Externalising and Total Behaviour Problems. Internalising Problems combines the Withdrawn, Somatic Complaints and Anxiety/ Depression sub constructs, while Externalising Problems combines the Delinquent and Aggressive Behaviour sub constructs.

**Results**: Results from CBCL/ 4-18 showed that all pupils from both groups had scores within the normal range for all constructs. However, results from the statistical analysis for comparison, Mann-Whitney U test, showed that pupils with CCVD scored significantly higher for Externalising Problems (U=697.50, p=0.02) and Total Behaviour Problems (U=647.00, p= 0.01). Significantly higher scores were observed in Withdrawn (U=714.00, p=0.02), Thought Problems (U=438.50, p<0.001) and Aggressive Behaviour (U=738.00, p=0.04). Odds ratios, 95% CI, showed significant relative risk for high Total Behaviour Problem (OR:2.39 ,CI:1.0-5.7), Externalising Problems (OR:2.32, CI:1.0-5.5), Withdrawn (OR:2.67, CI:1.1-6.5), Thought Problems (OR:9.64, CI:3.6-26.1) and Aggressive Behaviour (OR:10.26, CI:3.4-31.0) scores among pupils with CCVD.

**Conclusion**: Higher scores among CCVD pupils indicates that they present more behavioural and emotional problems compared to NCV pupils. Therefore, school vision screenings in Malaysia should also include colour vision to assist in the early clinical management of CCVD children.

## Introduction

Normal colour vision (NCV), in which, all of the three types of retinal cones are functioning well, is a vital attribute of visual perception. The importance of NCV is observed even in the early years of a person life as colour plays a vital role in teaching, which helps to improve memory and increase pupil’s interest in the learning process ( Bhoopal & Arya, 2016). However, the excessive use of colours in teaching and learning at primary schools may create confusion and a less favourable learning environment to pupils with congenital colour vision deficiency (CCVD), whose absorption spectra of cone pigments are defective.

A cross-sectional study conducted by Reddy & Hassan (2006) among 1214 primary school pupils aged between seven and 12 years old in Petaling Jaya, Malaysia, showed an overall CCVD prevalence rate of 2.60% with a significant male predominance of 4.80% and 0.20% for female. This indicates that in every classroom of 40 pupils, one or two male pupils is/are expected to have CCVD. In Malaysia, school vision screenings are conducted at primary one, which is at the age of 7. However, they do not provide screening for colour vision. This may cause pupils with CCVD to be unaware of their condition. Hence, they may not be able to adapt well with their surroundings, which may lead to behavioural, emotional and social issues. This may subsequently result in a decline in overall academic performance ( Espinda, 1971) as well as having a negative impact on the individual’s self-confidence ( Bailey, 2013).

The main objective of this case-control study was to compare behavioural and emotional issues among primary school pupils with CCVD (as case) and age, gender and class-matched pupils with normal colour vision (NCV) (as controls) in the Federal Territory of Kuala Lumpur, Malaysia using the Malay language ( *Bahasa Melayu*) adapted version of Child Behaviour Checklist/ages 4-18 (CBCL/ 4-18). The CBCL/ 4-18 is used to access behavioural and emotional problems (also known as behavioural syndromes). It has three broad groupings of syndromes scale: Internalising Problems, Externalising Problems and Total Problems. Internalising Problems combines the sub constructs of Withdrawn, Somatic Complaints, and Anxiety/Depression sub constructs, while Externalising problems combines the Delinquent Behaviour and Aggressive Behaviour sub constructs ( Achenbach, 1991). The Total Problems combines the scores of all the sub constructs ( Brown & Achenbach, 1992). The Child Behaviour Checklist/ages 4-18 (CBCL/ 4-18) has been translated and validated into 75 languages, and it is widely used for clinical diagnosis and in research ( Ivanova *et al.*, 2010). To date this questionnaire has not been used to study the behavioural and emotional problems among pupils with CCVD. The CBCL/ 4-18 has been widely used previously in various studies among children in Malaysia, and it was found that this checklist was a good screening tool for the maladjusted ( Normah & Shalisah, 1999; Talib *et al.*, 2011; Teoh, 2010). Studying the significance of this issue is important to assist in the early clinical management such as adaptive strategies and early counselling for the children with CCVD.

## Methods

The study is reported in accordance with the STROBE case-control reporting guidelines ( von Elm *et al.*, 2007).

### Study design

A case-control research survey using a parent-completed questionnaire was used to carry out this study.

### Study location

Ten national primary schools were selected by means of simple random sampling method from a list primary schools in four districts in the Federal Territory of Kuala Lumpur, Malaysia which are Kuala Lumpur (n=4), Sentul (n=2), Cheras (n=2) and Sungai Besi (n=2).

### Participant recruitment

In the beginning, we did not specify which parent should complete the CBCL/ 4-18, but the returned consent form were mostly fill out by mother of the pupils (n=1655, 95.6%). Thus, based on majority, we decided to recruit only mother of pupils as respondents in this study. A purposive sampling method was used whereby the participants were recruited based on particular consideration that is, the mother of pupils with CCVD, and age, gender and class-matched pupils with NCV ( Etikan, 2016). Inclusion and exclusion criteria in this study includes voluntary participation from mothers of pupils with CCVD and NCV (control group), pupils are between the age of eight and 11 years, and should have no other vision, physical, or cognitive disability.

### Sample size calculation

The required sample size of pupil with CCVD in this study was calculated based on the prevalence rate of CCVD among the population of primary school pupil in Petaling Jaya, Selangor which is at 2.60% ( Reddy & Hassan, 2006). Hence, the required sample size was calculated using Morgan’s simple sample size calculation formula ( Daniel, 1999).

$\begin{array}{l} \text{n}\;=\frac{{Ζ}^{2}\text{P(1-P)}}{{\text{d}}^{\text{2}}}\;\\ \text{n}\;=\;\frac{1.96^{\text{2}}(0.026)(1-0.026)}{0.05^{\text{2}}}\\ \text{n}\;=\;38.91\\ \text{n}\;\approx 40 \end{array}$

Where, Z is the standard value for level of confidence at 95.00% (1.96); P is the prevalence rate of primary school pupil with CCVD in Petaling Jaya, Malaysia at 2.60% and d is the margin of error set at 5.00%.

The required sample size was 40 primary school pupils from each group. Taking into consideration of dropouts, an additional 10% (4 pupils) was added to the sample size calculation. Thus, making the actual sample size to 44 pupils with CCVD. A total of 1732 (male=849 (49.0%), female=883 (51.0%) primary school pupils underwent the colour vision screening. The screening revealed a total of 46 (2.7%) pupils had CCVD. The prevalence rate of CCVD is higher in males (5.3%, n=45) than females (0.1%, n=1). The participants of this study consisted of mother of the pupils with CCVD (case group) and NCV as a control group. Based on the sample size calculation, only 44 mothers of pupils with CCVD (all male) who gave consent and agreed to participate in this study were recruited. The control group was equal to the size of the cases (case:control ratio of 1:1). Therefore, the control group included 44 mothers of pupils with NCV, where all the pupils that were age, gender and class-matched were selected by means of purposive sampling based their presence at the study location. All the pupils were given written consent to obtain their mother’s agreement to be recruited in this study. 

### Instruments

The questionnaire used in this study was the adapted Malay language version of the Child Behaviour Checklist/ages 4-18 (CBCL/ 4-18) completed by the mothers of pupils with CCVD and NCV to illustrate their children’s behavioural and emotional issues ( Achenbach & Rescorla, 2001) (Questionnaire is available from the Achenbach System of Empirically Based Assessment (ASEBA) website). This questionnaire comprised of 113 questions measuring eight sub constructs: Withdrawn, Somatic Complaints, Anxiety/Depression, Social Problems, Thought Problems, Attention Problems, Delinquent Behaviour and Aggressive Behaviour ( Achenbach, 1991). Respondents read and gave their views using a three-point Likert scale: scale of '0' indicated 'not true', the '1' scale indicated 'sometimes true’ and the ‘2’ scale indicated ‘very true’. This version, designed for 4 to 18 year old children, had clear instructions, and the mother of the pupils could complete it within 15 to 20 minutes without the need for supervision or guidance from the researcher.

### Procedure

Consent forms and information sheets were given to all pupils to obtain their parent’s consent to allow them to participate in this study. Only those with their parent’s consent were recruited. Participant’s parents were informed that all collected data would remain confidential.

Firstly, visual acuity and colour vision screening were conducted by the researcher at the selected schools from February 2018 till May 2018 to identify pupils with CCVD. Pupils who passed the inclusion criteria, underwent the visual acuity measurement which was conducted at both distant (6 metres) and near (40 cm) using the Early Treatment Diabetic Retinopathy Study (EDTRS) chart and colour vision screening using Ishihara 24-Pseudoisochromatic Plates (Kanehara Trading Co. Ltd, Tokyo, Japan) and followed by Farnsworth D-15 test (Munsell Color Company, Inc., Baltimore, MD, USA). The Ishihara 24-plates were performed by holding them under daylight at a distance of 50 cm and tilted so that each plate was at right angles to the line of vision. The time allocated to read each plate was less than 5 seconds. Those who failed to read four or more plates, were then asked to arrange 15 coloured caps on the Farnsworth D-15 test. To perform this test, pupils were instructed to arrange 15 randomly ordered coloured caps in order of hue with a reference coloured cap placed at the starting point for them to arrange the rest of the caps in an order. When the pupil had completed the test, the cap sequence is plotted and based on the number and direction of major crossovers on the plot, the type of colour vision deficiency were determined.

**Figure 1.**
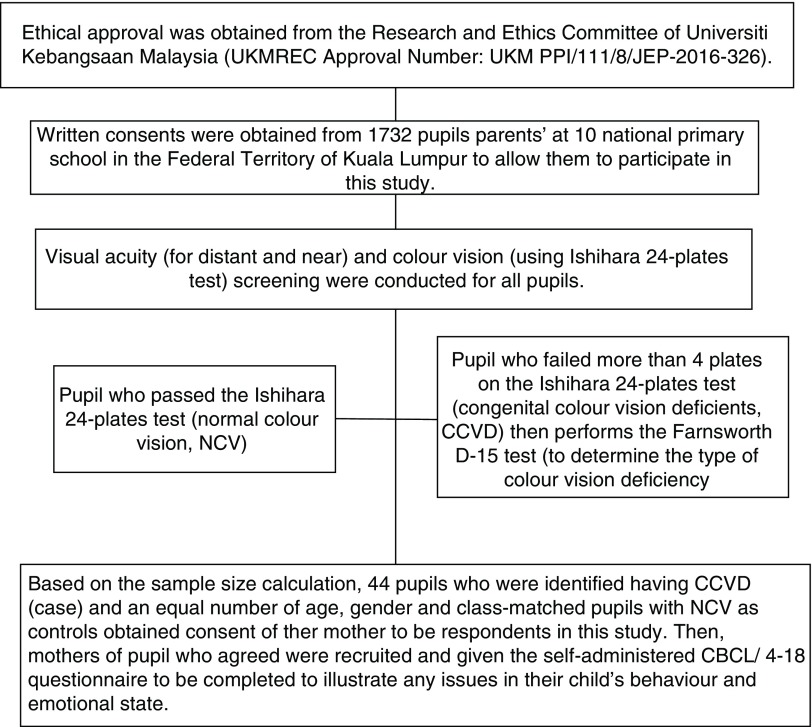
Flow diagram of participant recruitment procedure.

Then, pupils who were identified having CCVD and an equal number of age, gender and class-matched pupils with NCV were required to obtain consent of their mother to be respondents in this study. Only mothers of pupil who agreed were recruited and given the self-administered CBCL/ 4-18 questionnaire to be completed to illustrate any issues in their child’s behaviour and emotional state. Demographic data of all the pupils, which included age and type of colour vision deficiency (for pupil with CCVD), as well as their mothers’ which included age, race, education level, family income (monthly), marital status, medical and ocular history of the child, and awareness of their child’s colour vision problem were also recorded. A flow diagram of the participant recruitment procedure is as shown in Figure 1.

### Statistical analysis

The CBCL/ 4-18 template for hand-scoring was used to transfer of data from the questionnaire forms completed by the respondents to the hand-scored problem scales profile for the pupil. The raw scores for each sub construct are converted to age-standardized T-scores with the aid of the template to statistically analyse the data (T-score, *µ* = 50 and *σ* = 10). The quantitative data collected was analysed using the IBM SPSS Statistical 22.0 software.

The data was analysed for normality using the Shapiro-Wilks test, and the Descriptive Statistics test was carried out to measure the behaviour and emotional of the pupil by looking at the skewness of the graph, whether positive or negative. The tests revealed that the data was not normally distributed. Thereafter, the non-parametric statistical analysis, Mann-Whitney U test, was used to compare and determine whether there was a significant difference in behaviour and emotions between pupils with CCVD (case) and with NCV (control). Odds ratios were also calculated with 95% confidence intervals (CI) as estimates of the relative risk for high symptom scores among children with CCVD compared with the control group ( Liljenfeldt & Pettersson, 2017). A two-tailed p value below 0.05 was considered statistically significant.

## Results

The demographic data of mothers and their children with CCVD and NCV who agreed to be recruited as respondents in this study are shown in Table 1. The majority (86.36% of mothers of pupils with CCVD, and 72.72% of mother of pupils with NCV), were within 31 to 50 years old and mostly are married. About 45.45% of mother of pupils with CCVD and 38.64% of mother of pupils with NCV had undergraduate degrees, and most of them had a monthly family income of RM (Malaysian ringgit) 4,001-RM 6,000. Only 15.91% of mothers of pupils with CCVD stated that they were aware of their child’s condition. All aged-matched pupil in both the CCVD group and NCV group were within the age group of 8 to 11 years old (µ =9.47, σ =1.04). Among those with CCVD, 33 (75.00%) were identified as deuteranomalous trichromats and 11 (25.00%) as protanomalous trichromats. All mother of pupils with CCVD and NCV ruled out any known medical and ocular history of the child.

**Table 1.** Demography data of all the participants (mother of pupils) and pupils (case and controls).

Demography		Description	N	Percentage (%)
1. Mother of pupil				
Age (years)	CCVD	18–30 31–50 >51	6 38 0	13.64 86.36 0.00
	NCV	18–30 31–50 >51	8 32 4	18.18 72.72 9.09
Race	CCVD	Malay Chinese	42 1 1	95.45 2.27 2.27
	NCV	Malay Chinese Indian	42 1 1	95.45 2.27 2.27
Marital status	CCVD	Married	43 1	97.73 2.27
	NCV	Married Single Parent	42 2	95.45 4.55
Education Level	CCVD	Primary Secondary Diploma Undergraduate Degree Master Degree	3 13 6 20 2	6.82 29.55 13.64 45.45 4.55
	NCV	Primary Secondary Diploma Undergraduate Degree Master Degree	4 16 4 17 3	9.09 36.36 9.09 38.64 6.82
Family income (monthly)	CCVD	<RM 4,000 RM 4,001–RM 6,000 RM 6,001–RM 8,000 >RM 8,001	10 29 4 1	22.73 65.91 9.09 2.27
	NCV	<RM 4,000 RM 4,001–RM 6,000 RM 6,001-RM 8,000 >RM 8,001	5 34 3 2	11.36 77.27 6.82 4.55
Awareness of child’s colour vision problem	CCVD	Yes No	7 37	15.91 84.09
	NCV	Yes No	0 44	0.00 100.00
2. Pupils				
Age (years)	CCVD	8 9 10 11	9 15 11 9	20.45 34.09 25.00 20.45
	NCV	8 9 10 11	9 15 11 9	20.45 34.09 25.00 20.45
Type of CCVD (only for pupil with CCVD)		Protanomalous Trichromacy Deuteranomalous Trichromacy	11 33	25.00 75.00

CCVD – congenital colour vision deficiency, NCV – normal colour vision

**Figure 2.**
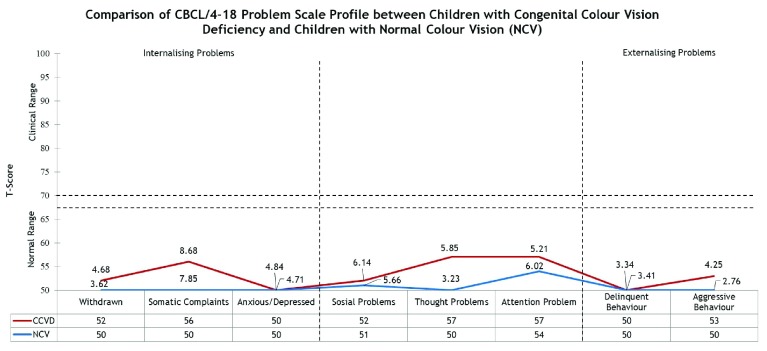
Comparison of Child Behaviour Checklist (CBCL)/ 4-18 Problem Scale Profile for pupils with congenital colour vision deficiency (CCVD) and normal colour vision (NCV). Horizontal broken lines = Borderline clinical range.

A normality test was conducted on each sub construct to determine the distribution of data. The Shapiro-Wilk, W, test for all eight sub constructs and combination of sub constructs, revealed a p-value <0.05 which shows that all data were not normally distributed. Descriptive statistical analysis results for the CBCL/ 4-18 questionnaire are presented based on raw score which had been converted into standard scores (T-score, *µ* =50 and *σ* =10). The conversion to T-score allows the comparison of scores obtained with normative data from other pupils of the same age range as shown in Table 2 ( Achenbach, 1991). Based on the frequency analysis, the scores for all eight sub constructs and the three broad groupings of behavioural syndrome were within the normal range of both groups as shown in Figure 2.

**Table 2.** Classification of normal clinical range values for Child Behaviour Checklist (CBCL)/4-18 syndrome scale.

T-score	Range
1. Sub constructs (Eight sub constructs)	
< 67	Normal
67 – 70	Borderline clinical
> 70	Clinical
2. Combination of sub constructs (Three broad groupings of syndrome)	
< 60	Normal
60 – 63	Borderline clinical
> 63	Clinical

As the data were not normally distributed, non-parametric statistical analysis, Mann-Whitney U test, was chosen to compare the results for both groups. As illustrated in Table 3, significantly higher scores were observed for the CCVD group in Externalising Problems (U=697.50, p=0.02) and Total Behaviour Problems (U=647.00, p=0.01). Similarly, significantly higher scores for the CCVD group were also observed in the sub constructs Withdrawn (U=714.00, p=0.02), Thought Problems (U=438.50, p< 0.001) and Aggressive Behaviour (U=738.00, p=0.04).

**Table 3.** Results obtained from Mann-Whitney U test analysis.

Scale	Group	Mean Rank	U	p-value
Withdrawn	CCVD NCV	50.27 38.73	714.00	0.02
Somatic Complaints	CCVD NCV	48.49 40.51	792.50	0.13
Anxious/Depressed	CCVD NCV	46.57 42.43	877.00	0.40
Social Problems	CCVD NCV	48.43 40.57	795.00	0.13
Thought Problems	CCVD NCV	56.53 32.47	438.50	<0.001
Attention Problems	CCVD NCV	46.19 42.81	893.50	0.53
Delinquent Behaviour	CCVD NCV	47.09 41.91	854.00	0.27
Aggressive Behaviour	CCVD NCV	49.73 39.27	738.00	0.04
Internalising Problems	CCVD NCV	48.30 40.70	801.00	0.16
Externalising Problems	CCVD NCV	50.65 38.35	697.50	0.02
Total Problems	CCVD NCV	51.80 37.20	647.00	0.01

CCVD – congenital colour vision deficiency, NCV – normal colour vision

Subsequently, odds ratios were calculated with 95% confidence intervals (CI) as estimates of the relative risk for high Total Behaviour Problem, Externalising Problems, Withdrawn, Thought Problems and Aggressive Behaviour scores among children with CCVD compared with the control group. When a cut-off was applied at the T-score ≥60, which indicates borderline clinical behavioural syndrome, odds for high scores on the Total Problems scale was 2.39(CI 1.0-5.7), Externalising Problems was 2.32 (CI 1.0-5.5), Withdrawn was 2.67(CI 1.1-6.5), Thought Problems 9.64 (CI 3.6-26.1) and Aggressive Behaviour was 10.26 (CI 3.4-31.0) as shown in Table 4.

**Table 4.** Results obtained from odds ratio (OR) with 95% confidence intervals (CI) of high scores (i.e. T-score ≥60 indicating borderline clinical syndrome) on summary scales of Child Behaviour Checklist (CBCL)/ 4-18 in pupils with congenital colour vision deficiency (CCVD) compared with control group.

	Total Problem				Externalising Problem			Withdrawn			Thought Problems			Aggressive Behaviour		
	T-score <60 (%)	T-score ≥60 (%)	OR (CI)/ *p*-value	T-score <60 (%)	T-score ≥60 (%)	OR (CI)/ *p*-value	T-score <60 (%)	T-score ≥60 (%)	OR (CI)/ *p*-value	T-score <60 (%)	T-score ≥60 (%)	OR (CI)/ *p*-value	T-score <60 (%)	T-score ≥60 (%)	OR (CI)/ *p*-value
CCVD	31 (72.09)	12 (27.91)	2.39 (1.0- 5.7)/ .040	24 (55.81)	19 (44.19)	2.32 (1.0-5.5)/ .043	22 (51.16)	21 (48.84)	2.67 (1.1-6.5)/ .024	30 (69.77)	13 (30.23)	9.64 (3.6- 26.1)/ < .001	39 (90.70)	4 (9.30)	10.26 (3.4-31.0)/ < .001
NCV	22 (51.16)	21 (48.84)		15 (34.88)	28 (28.65)		12 (27.91)	31 (72.09)		8 (18.60)	35 (81.40)		19 (44.19)	24 (55.81)	

NCV – normal colour vision

## Discussion

This study compares behavioural and emotional issues among primary school pupils with CCVD and NCV. The results from this study suggest that pupils with CCVD presented more behavioural and emotional problems as compared to NCV pupils. It is found that CCVD pupils might be at a higher risk of developing social and attention problems. Pupils with CCVD having high scores for sub construct of Withdrawn tend to present with behaviours such as preference to be alone, shy, staring blankly and show signs of sadness. Socially withdrawn pupil aged above 7 years, often encounter problems in social interactions with peers and social skills ( Fink *et al.*, 2015). Moreover, with increasing age social withdrawal becomes accompanied by feelings of loneliness and depression ( Matthews *et al.*, 2015). This may contribute to acting-out behavior in form of social aggression that are associated with behaviours such as arguing, screaming, showing off, attention-seeking, bragging, teasing, being demanding, threatening behaviour and being temperamental. Furthermore, pupils’ class participation and social skills is an important contributor to their academic competence ( Rabiner *et al.*, 2016). Additionally, pupils with CCVD having a combination of social withdrawal, aggressive behavior, along with thought problems with characteristics of obsessing on certain thoughts, finding it difficult to concentrate, staring blanking and having strange ideas or behaviours, can diminish the ability to learn, which affects academic performance ( Rhoades *et al.*, 2011).

Our finding revealed that pupils with CCVD are experiencing more internalising problems. There is a possibility that in general, people are better at recognising pupil’s externalising problems, such as aggression, internalising problems such as depression and social withdrawal. This is because externalising problems may be more likely to induce a sense of worry in the people surrounding them, while the internalising problems faced by these pupils may be overlooked by their parents and teachers who are unaware of the pupil’s colour vision impairment. This problem can be overcome by an early colour vision screening. For screening, the Ishihara test is widely used as it is quick and easy to administer, inexpensive, and has a high validity ( Birch, 1997). Though, in a recent published review article on ‘Is screening for congenital colour vision deficiency in school students worthwhile?’, Ramachandran & colleagues (2014) stated that there’s minimal evidence to support the screening for CCVD in school. However, this article has received disagreement from other researchers. Based on personal experience with congenital colour vision defects, Cole (2015) agree to disagree with the conclusion made by the writer. He believes that pupils with CCVD do need to know about their condition before the end of their schooling. This is also supported by Long & colleagues (2015) as they believe that delaying the diagnosis and awareness of CCVD may create significant emotional and psychological impact. Based on their clinical perspective of working with young adults having CCVD, they see many of them who comes for a comprehensive colour vision examination for job or tertiary education recruitment, often gets shocked which accompanied with grief, disbelief and anger upon discovering their condition. Thus, an early colour vision screening is school would be the best option. This will enable useful early counselling and adaptive strategies to be implemented especially in classrooms as early as possible.

Classroom behaviour is very important in primary school life and pupils who display problematic behaviours also tend to have deficits in social and emotional skills ( Wagner & Ruch, 2015). Besides that, behavioural problems present in early childhood may develop into greater problems in later life ( Ogundele, 2018). It is therefore important to broaden the age range of the current study in order to take into account mental health problems across the different stages of childhood development such as preschool and secondary school. Research has found generally pupils’ behavioural problems differ according to gender. Previous studies conducted among pupils from various cultures have found that male pupils are more likely to achieve higher scores on Externalising Problems as compared to Internalising Problems ( Achenbach *et al.*, 1990; Achenbach *et al.*, 1990).

A limitation of this study was having no data from female pupils with CCVD due to the very low prevalence rate. Thus, in the present study, CBCL/ 4-18 data was only collected from age and class-matched male pupils in both case and control groups for comparison. Therefore, it is not possible to compare scores between male and female pupils. However, this study showed that male pupils with CCVD had significantly higher scores as compared to the male pupils in the control group with NCV. Further understanding of the behavioural problems among female pupils with CCVD is recommended for future studies. Our findings should also be viewed in the context of some methodological limitations. Because this study was conducted in national primary schools in Malaysia, most pupils with CCVD were mainly Malay with only one Chinese and one Indian. Thus, our findings may not generalise to minorities. Future studies are to be conducted in vernacular schools.

## Conclusion

In conclusion, the analysis of the CBCL/ 4-18 showed that the scores for all problem sub constructs obtained by pupils with CCVD were within the normal range. However, their scores were higher than of their peers with NCV, which suggest that the pupils with CCVD present more behavioural and emotional problems as compared to NCV pupils. These findings provide important new data on the behavioural and emotional problems of Malaysian primary school pupils with CCVD. This study emphasises the importance of additional studies to be conducted to understand this issue in depth which provides insight to assist in the clinical management of the CCVD children. Thus, early school visual screening in Malaysia should also include colour vision so that the child, their family, and school teachers are aware of their condition as early as possible to ensure the well-being of the child.

## Data availability

Harvard Dataverse: Dataset 1. Demographic Data & CBCL/4-18 Scores-Behavioural and Emotional Issues among Primary School Pupils with Congenital Colour Vision Deficiency in Federal Territory of Kuala Lumpur, Malaysia. https://doi.org/10.7910/DVN/DPZHI4 ( Thomas *et al.*, 2018)

## Ethics approval

Ethical approval for this study was obtained from the Research and Ethics Committee of Universiti Kebangsaan Malaysia (UKMREC Approval Number: UKM PPI/111/8/JEP-2016-326).

## Consent

Written informed consent for publication of the participants details were obtained from the participants.
