# Supplementary material for: Behavioural and emotional issues among primary school pupils with congenital colour vision deficiency in the Federal Territory of Kuala Lumpur, Malaysia: A case-control study
Source: F1000Res. 2018 Nov 21;7:1834. [Version 1] doi: 10.12688/f1000research.17006.1 (PMC6372925; doi:10.12688/f1000research.17006.1)
Supplement: Peer review history

# Peer review: Referee response for version 1

**DOI:** 10.5256/f1000research.18594.r42106

**Published:** 2019-02-08

**Authors:** Wan-Hitam Wan-Hazabbah

**Affiliations:** 1 Department of Ophthalmology, School of Medical Sciences, Universiti Sains Malaysia, Kubang Kerian, Malaysia

Overall, this is a good study. However, the study only involved mothers of the children to answer the questionnaires. The researchers should also consider interviewing the teachers because nowadays the students spend more of their time in school compared to home. In certain circumstances the mothers may not know what actually happened at school. The teachers may know more of the achievements and problems of the children.

- Title - Clearly represents the study.
- Abstract - Satisfactory.
- Introduction - Good. Able to highlight the importance of the study.
- Objective - Good. Clearly stated.
- Methodology - Very good. Clearly elaborate.
- Results - Well presented.
- Discussion - Good coverage.
- Limitation - Well addressed.
- Conclusion - Good. Answered the objective of the study.
- References - Good. Up to date.
- Tables & Figures - Clearly stated results.

I have read this submission. I believe that I have an appropriate level of expertise to confirm that it is of an acceptable scientific standard.

# Peer review: Referee response for version 1

**DOI:** 10.5256/f1000research.18594.r43676

**Published:** 2019-02-06

**Authors:** Stephen J. Dain

**Affiliations:** 1 School of Optometry and Vision Science, The University of New South Wales, Sydney, New South Wales, Australia

This is an interesting report in a little studied area. It would benefit from a little wider literature review, better characterisation of the CCVD subjects and attention to the multiple tests nature of the analysis.

**Introduction:**

- Paragraph 1, Last line: Absorption spectra are not really “defective”. They are altered.
- See Bacon (1971 ^1^). It would also be useful to show that you have read Steward and Cole (1989 ^2^) and Cole *et al.*, (2002 ^3^).
- Paragraph 2, Line 10: And their parents.

**Methods:**

- Recruitment, Line 8: Were the pupils also matched for school (as a proxy for socio-economic matching)? This may also be addressed in Procedure paragraph 3 where you say that they were “class-matched”. Is that also implying school matched?
- Procedure: Was there an inclusion/exclusion criterion for VA?
- Procedure, paragraph 2: Was the pass/fail (4 errors) applied to all plates, screening and diagnostic, numerical and tracing?  In reality it should only be the screening plates. Daylight – artificial or natural? If artificial, details of source, correlated colour temperature and general colour rendering index. If natural, from which direction? Not that this matters as much at the latitude of KL. Illuminance level?

**Results:**

- Was any correction for multiple comparisons made?
- How did you decide if they were deuteranomalous or protanomalous? Strange that there were no dichromats. Or was it that you did not use an anomaloscope and could not actually identify dichromats? In which case you should talk deutans and protans. See also Table 2. Its a pity. Relating the problems to the degree of deficiency would be very informative. Do the dichromats, and strongly anomalous, who generally know, cope better than those who do not or worse because they are more affected?

**Discussion:**

- Last paragraph: I don’t see the lack of a female data as a limitation. In colour vision it is a fact of life. Also the female CCVD are predominantly deutan and less affected (manifesting the lesser of the two deficiencies that they carry).

I have read this submission. I believe that I have an appropriate level of expertise to confirm that it is of an acceptable scientific standard, however I have significant reservations, as outlined above.

## References

[1] BaconL: Colour Vision Defect – an Educational Handicap. *Med. Officer*.1971;125(16) :199-209

[2] StewardJMColeBL: What do color vision defectives say about everyday tasks?. *Optom Vis Sci*.1989;66(5) :288-95 278749210.1097/00006324-198905000-00006

[3] ColePMBruschiCJTamangBL: Cultural differences in children's emotional reactions to difficult situations. *Child Dev*.73(3) :983-96 1203856410.1111/1467-8624.00451

# Peer review: Referee response for version 1

**DOI:** 10.5256/f1000research.18594.r40971

**Published:** 2019-01-07

**Authors:** Khairidzan Mohd Kamal

**Affiliations:** 1 Department of Ophthalmology, Kulliyyah of Medicine, International Islamic University Malaysia (IIUM), Kuantan, Malaysia

This is a good article and research work. It addresses the question of whether congenital colour vision deficiency (CCVD) affects the behavioural and emotional status of school children at the early ages. This is an especially important question to explore given that the current teaching and learning methods in primary schools utilize significant animated and graphically based materials. The researchers have chosen a comprehensive screening method which involved only mainstream education systems (public schools) and adopted standard questionnaires with translation (CBCL/4-18) to be given to parents. The authors have employed appropriate statistical tools for normality and non-parametric analysis. Matching processes between affected children with CCVD with control groups were well performed which allowed the researchers to analyse their background and economic status. For example, education background and household income for each group were compared to show the possibility of these two factors in the children’s behavioural and emotional status. However, since the recruitment was done in schools around metropolitan areas with standardized teaching facilities, it would be difficult to conclude whether this result is applicable to school children with CCVD in the rural area.

 The discussions and conclusions highlighted that children with CCVD were within the normal range but showed significantly higher scores than children without CCVD, Externalising and Total Behavioural Problems. It is more important to note that students in this study came from public school systems with household income that represents borderline urban poverty line. Low awareness rate among parents of both affected and non-affected children is alarming and reasons for this should be highlighted in the article. It would be interesting to find out whether children from affluent backgrounds would have the same results as revealed in this research work.

I have read this submission. I believe that I have an appropriate level of expertise to confirm that it is of an acceptable scientific standard.
